# Detecting differentially methylated regions using a fast wavelet-based approach to functional association analysis

**DOI:** 10.1186/s12859-021-03979-y

**Published:** 2021-02-10

**Authors:** William R. P. Denault, Astanand Jugessur

**Affiliations:** 1 Department of Genetics and Bioinformatics, Norwegian Institute of Public Health, Oslo, Norway; 2 Centre for Fertility and Health, Norwegian Institute of Public Health, Oslo, Norway; 3 Department of Global Public Health and Primary Care, University of Bergen, Bergen, Norway

**Keywords:** Wavelets, DNA methylation, EWAS, Association analysis, Epigenetics

## Abstract

**Background:**

We present here a computational shortcut to improve a powerful wavelet-based method by Shim and Stephens (Ann Appl Stat 9(2):665–686, 2015. 10.1214/14-AOAS776) called WaveQTL that was originally designed to identify DNase I hypersensitivity quantitative trait loci (dsQTL).

**Results:**

WaveQTL relies on permutations to evaluate the significance of an association. We applied a recent method by Zhou and Guan (J Am Stat Assoc 113(523):1362–1371, 2017. 10.1080/01621459.2017.1328361) to boost computational speed, which involves calculating the distribution of Bayes factors and estimating the significance of an association by simulations rather than permutations. We called this simulation-based approach “fast functional wavelet” (FFW), and tested it on a publicly available DNA methylation (DNAm) dataset on colorectal cancer. The simulations confirmed a substantial gain in computational speed compared to the permutation-based approach in WaveQTL. Furthermore, we show that FFW controls the type I error satisfactorily and has good power for detecting differentially methylated regions.

**Conclusions:**

Our approach has broad utility and can be applied to detect associations between different types of functions and phenotypes. As more and more DNAm datasets are being made available through public repositories, an attractive application of FFW would be to re-analyze these data and identify associations that might have been missed by previous efforts. The full R package for FFW is freely available at GitHub https://github.com/william-denault/ffw.

## Background

Despite the recent surge of interest in functional association analysis of various types of high-dimensional data, e.g., those from biomechanical research [1], quantitative trait loci (QTL) mapping [2], genome-wide association studies (GWASes) [3], and epigenome-wide association studies (EWASes) [4, 5], the majority of genome-wide screenings are still largely based on testing one SNP or one CpG at a time (single-point analysis). Single-point analyses are limited because they incur a substantial multiple-testing burden and ignore the genomic context of the association. Bump-hunting methods [4, 6] can, to some extent, alleviate these issues by detecting a predetermined difference in the spatially ordered variables (e.g., SNPs or CpGs) that correlate with the trait or disease under scrutiny. However, as the name implies, bump-hunting methods can only analyze a single bump at a time, failing to consider the combined effect of multiple bumps in a given region. Besides the lack of power [5], bump-hunting methods also rely on a re-sampling procedure, such as bootstrapping or permutation, which makes them computationally more intensive (for details, see [4]).

To help address these methodological shortcomings, we developed a wavelet-based method to enable the detection of more complex signals than those present in a single bump. Although several methods are now available for functional association analysis based on wavelets [5, 7–9], they do not scale well in terms of computational time when the sample size (*n*) or the number of regions (*R*) becomes exceedingly large (e.g., when $n\approx 1000$ or $R > 1000$). As wavelet coefficients are not independent, Lee and Morris [5] and Ma and Soriano [9] proposed searching for associations between a trait and a function by using wavelet regression that takes into account the dependencies between the wavelet coefficients. However, this requires the use of a re-sampling procedure such as Markov Chain Monte Carlo (MCMC) (exemplified in [5, 7]) or the computation of complex analytical posterior distributions as in Ma and Soriano [9]. To address these issues, Shim and Stephens [8] proposed simplifying the modeling by omitting the dependencies and using a likelihood ratio test to search for associations between a trait and a function. This simplification is, however, still limited because of the need for permutations to evaluate the significance of the likelihood ratio.

In our current approach, which we call “Fast Functional Wavelet” (FFW), we combine the framework of Shim and Stephens [8] with recent results on the theoretical null distribution of Bayes factors by Zhou and Guan [10]. Our approach allows a fast emulation of the test statistic in Shim and Stephens [8] and reduces the computational time considerably. The main difference between FFW and WaveQTL is that FFW requires regressing the trait of interest on the wavelet coefficients, regardless of the application. Hence, the design matrix for association is kept constant across all the screened regions. This simple modification enables the null distribution of the test statistic to be simulated using only a $\chi ^2 _1$ distribution, thereby circumventing the need for extensive permutations to assess the significance of a region. By keeping the design matrix for association constant across the screened regions, and using the results of Zhou and Guan [10], we can show that the same distribution can be used to emulate the null distribution of each regional test. This null distribution depends on a single parameter that is easily computed. Keeping the design matrix constant across the screened regions can lead to a reverse regression, resulting in shrunken estimates and a potential loss of power (see Fuller, Chapter 1 [11]). Besides making the null distribution easier to emulate, reverse regression also allows the analysis of a wider variety of traits (continuous, binary, or count).

Although the focus of the current paper is on DNAm data, we describe FFW more broadly to highlight its utility for other types of high-dimensional data, such as those from a GWAS [3], dsQTL analysis [2], and functional data from biomechanical research [1].

## Description of our wavelet-based approach

There are different types of wavelets, and readers interested in a more comprehensive introduction to wavelets are referred to Nason [12]. In our application here (FFW), we only consider the simplest type of wavelet—the Haar wavelet.

### Processing

For simplicity, we consider genetic regions composed of evenly-spaced variables of length $2^J$. As the assumption of evenly-spaced variables rarely holds in practice, we use the approach of Kovac and Silverman [13] to handle this limitation in our software implementation. Their approach mainly consists of using an interpolation on the observed data to obtain evenly-spaced variables of length $2^J$.

A given region is defined as a compound of $X_1,\ldots X_{2^J}$, with $2^J$ spatially-ordered variables. Suppose we observe these $2^J$ variables for *n* individuals; we then denote $X_{i,k}$ as the *k*th observation of the *i*th variable. The wavelet coefficients for the individual *k* are defined as follows. For wavelet coefficients corresponding to the highest scale or resolution (i.e., *J*), and for $i \in \llbracket 1,2^{J-1}\rrbracket$

$$\begin{aligned} {\tilde{X}}_{J,i,k}= X_{2i,k} -X_{2i-1,k} \end{aligned}$$

These wavelet coefficients correspond to local differences between adjacent variables. For a lower scale (i.e., $j < J$), the corresponding wavelet coefficients are computed as follows:

$$\begin{aligned} {\tilde{X}}_{j,i,k}= \check{X}_{j+1,2i,k} -\check{X}_{j+1,2i-1,k} \end{aligned}$$

where $\check{X}_{j,2i,k}$, is defined as:

1 $$\begin{aligned} \check{X}_{j,2i,k} = {\left\{ \begin{array}{ll} \forall i \in \llbracket 1,2^{J-1}\rrbracket , X_{2i,k} + X_{2i-1,k}, &{} \hbox { if}\ j=J \\ \forall i \in \llbracket 1,2^{j-1}\rrbracket , \check{X}_{j+1,2i,k} + \check{X}_{j+1,2i-1,k}, &{} \hbox { if}\ 1\le j < J \end{array}\right. } \end{aligned}$$

$\check{X}_{j,2i,k}$ correspond to the scaled average of the adjacent variables for individual *k* (for further details, see Nason [14]). Finally, the wavelet coefficients for the lowest scale (i.e., 0) are computed as follows:

$$\begin{aligned} {\tilde{X}}_{0,1,k}= \sum _{i=1}^{2^J} X_{i,k} \end{aligned}$$

The procedure described above is known as Mallat’s pyramid algorithm for signal processing [15]. To ease comprehension, we denote ${\tilde{X}}_{jl}$ as the random variable representing the wavelet coefficient at the scale *j*, with $1 \le j \le J$, and at the location *l*, with $1 \le l \le 2^{j-1}$.

### Modeling

We first summarize the work of Shim and Stephens [8] before presenting our main methodological contributions in the “Significance of a region” section further below.

To identify associations between a region and a phenotype (denoted as $\Phi$ hereafter), we assess whether specific scales are associated with $\Phi$ at different locations. Let $\pi$ be a vector of length *J*, where $\forall j \in [0:J], \pi _j \in [0,1]$ and $\pi _j$ represents the proportion of wavelet coefficients at scale *j* associated with $\Phi$. To assess the significance of a given genetic region, we test the following hypothesis:

2 $$\begin{aligned} H_0:\pi =(0,\ldots ,0) vs H_1:\exists j \in [0:J], \pi _j \ne 0 \end{aligned}$$

In the next sections, we describe the test statistic (likelihood ratio), how its different components are computed, and how its significance is tested.

#### Bayes factors

To test for associations between $\Phi$ and the wavelet coefficient ${\tilde{X}}_{jl}$ for a given region, we use the Normal-Inverse-Gamma (NIG) prior to perform a regression between each wavelet coefficient and $\Phi$. Note that our framework easily allows confounders to be incorporated into the regression models. We quantile-transform each wavelet coefficient across the individuals to reduce the proportion of spurious associations due to distribution-related issues.

The association models for each scale and location are defined as follows:

3 $$\begin{aligned}&M_0: {\tilde{X}}_{jl} = \beta _{jl,0} +\beta _{jl,C}C+\epsilon \nonumber \\&M_1: {\tilde{X}}_{jl} = \beta _{jl,0} + \beta _{jl,1}\Phi +\beta _{jl,C}C+\epsilon \end{aligned}$$

where *C* is a matrix of dimension $c \times n$, $\beta _{jl,C}$ is a matrix of dimension $1 \times c$ and $\epsilon \sim N(0, \sigma ^2)$, where $\sigma ^2$ is unknown. Next, we compute the Bayes factors of the wavelet regression *jl* using the closed form provided by Zhou and Guan [10] for the NIG prior.

#### Ratio statistic

Our goal is to assess the significance of the vector $\pi = (\pi _0,\ldots , \pi _j,\ldots ,\pi _J)$, where $\pi _j$ represents the proportion of wavelet coefficients at scale *j* associated with $\Phi$, and ${\tilde{X}}$ is the wavelet representation of the individual functions. To test the significance of $\pi$, we construct a test statistic by computing the following likelihood ratio:

4 $$\begin{aligned} \Lambda (\pi , {\tilde{X}}, \Phi ) = \frac{p( {\tilde{X}} |\pi , \Phi ) }{p( {\tilde{X}} |\pi \equiv 0, \Phi )} \end{aligned}$$

Following the approach of Shim and Stephens [8], we denote $\gamma _{jl}$ as the random variable with support $\{0,1\}$. Thus, $\gamma _{jl}=1$ if the wavelet coefficient ${\tilde{X}}_{jl}$ is associated with $\Phi$, and 0 if not. We consider $\pi$ as a hyperparameter of $\gamma _{jl}$; i.e.,

5 $$\begin{aligned} p(\gamma _{jl} =1| \pi ) = \pi _j \end{aligned}$$

Shim and Stephens [8] assume independence between the wavelet coefficients. However, this may not to hold in practice [16]. Under the assumption of independence of the wavelet coefficients, we can rewrite 4 as:

6 $$\begin{aligned} \Lambda (\pi , {\tilde{x}}, \Phi )&= \prod _{j,l} \frac{p( {\tilde{x}}_{jl} |\pi _j, \Phi ) }{p( {\tilde{x}}_{jl} |\pi _j = 0, \Phi )} \end{aligned}$$

7 $$\begin{aligned}&= \prod _{j,l} \frac{\pi _j p( {\tilde{x}} |\gamma _{jl}=1, \Phi ) + (1-\pi _j) p( {\tilde{x}} |\gamma _{jl}=0, \Phi ) }{p( {\tilde{x}} |\gamma _{jl}=0, \Phi )} \end{aligned}$$

We denote $BF_{jl} ({\tilde{x}},\Phi ) = \frac{p( {\tilde{x}}_{jl} |\gamma _{jl}=1, \Phi ) }{p( {\tilde{x}}_{jl} |\gamma _{jl}= 0, \Phi )}$ as the Bayes factor of the association between the wavelet coefficient at scale *s* and location *l*. Using this notation, we can rewrite 7 as:

8 $$\begin{aligned} \Lambda (\pi , {\tilde{x}}, \Phi ) = \prod _{j,l} \left[ \pi _j BF_{jl} +(1-\pi _j)\right] \end{aligned}$$

We then compute the likelihood ratio statistic by maximizing the lambda statistics over $\pi$ and estimating ${\hat{\pi }}$ using the EM algorithm.

9 $$\begin{aligned} {\hat{\Lambda }} ({\tilde{x}}, \Phi ) = max_{ \pi \in [0,1]^J} \Lambda (\pi , {\tilde{x}}, \Phi ) \end{aligned}$$

#### Significance of a region

As the distribution of $\Lambda$ is unknown, we simulate $\Lambda$ under $H_0$ by simulating $BF_{jl}$ under $H_0$. Recently, Zhou and Guan [10] showed that, under $H_0$ and a wide spectrum of priors, the Bayes factors (including the NIG prior) follow a specific distribution for a Gaussian model. More precisely,

10 $$\begin{aligned} 2log(BF) = \lambda _1 Q_1+ log(1-\lambda _1)+\epsilon \end{aligned}$$

where $Q_1$ is a non-central chi-squared random variable with $df=1$, and $\epsilon = O(1)$ and its non-centrality parameter has a closed-form. The parameter $\lambda _1$ is the largest eigenvalue of $X\left( X^t X+ V_b ^{-1}\right) ^{-1}X^t$, where *X* is the design matrix. Specifically, $X= \left( {\mathbf {1}},Y, C^t\right)$ and $V_b=diag( \sigma _b ^2 )$ ($\sigma _b$ is the prior effect size of the NIG prior for the intercept and the covariates). By keeping the design matrix constant across the regions, $\lambda _1$ stays the same for all the regions and only needs to be computed once. The non-centrality parameter is region-dependent in general, but it is exactly zero when the null hypothesis of the Bayes factor is $\beta _{jl,1}=0$. Zhou and Guan [10] showed that, for *df* = 1, $Q_1$ is asymptotically equal to the likelihood ratio test statistic for Gaussian linear models. In other words, $Q_1$ is equal to a simple chi-squared statistic with one degree of freedom. Therefore, we use the approximation in Eq. (11) for the distribution of the Bayes factors. Note also that Zhou and Guan [10] showed that this approximation is exact when using a Normal prior.

11 $$\begin{aligned} 2log(BF) \approx \lambda _1 \chi ^2_1+ log(1-\lambda _1) \end{aligned}$$

By using this approximation, it is only necessary to compute a single parameter for all the regions. We can then perform *M* independent simulations of the vector of Bayes factors under $H_0$. This corresponds to *M* vectors of length $2^{J}$, corresponding to the number of wavelet coefficients. Next, for each simulated vector of Bayes factors $BF_m$, we compute the simulated likelihood ratio ${\hat{\Lambda }}_{ m } = max_{ \pi \in [0,1]^J} \Lambda (\pi ,BF_m)$ using the procedure described above. Monte Carlo methods for p value estimation can then be applied to the set of observed statistics.

## Simulations and application

We performed a set of simulations to evaluate the gain in computational time with FFW and to assess the significance of the test statistic. We also evaluated the statistical power of FFW using a realistically simulated dataset. Lastly, we ran a sensitivity analysis for the priors to assess the sensitivity of FFW according to the choice of prior. All the simulations were performed on an ordinary laptop, equipped with an Intel(R) i7-700HQ 2.80 GHz processor and 8 GB of RAM.

### Gain in computational time

**Fig. 1:**
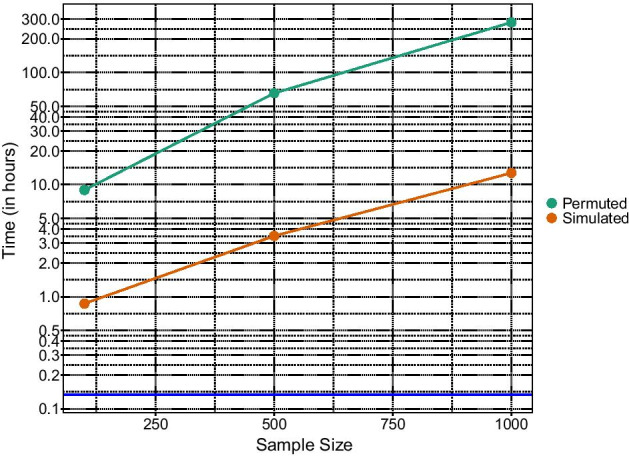
Comparison of the computational time under $H_0$ when performing an EWAS with FFW and WaveQTL for different sample sizes. The y-axis has been log-transformed. The green curve indicates the computational time using the permutation procedure described by Besag and Clifford [29], which is based on a maximum of 10 permutations being larger than the observed test statistics (the set up is according to Shim and Stephens [8]). The orange curve indicates the computational time using FFW. The blue line indicates the computational time of the simulations required in FFW, which remains constant with increasing sample size

We performed separate EWASes using FFW and WaveQTL, and report the run time for different sample sizes. Figure 1 illustrates the substantial gain in computational time with FFW. The green and orange curves represent the total time it took to perform an EWAS based on DNAm data generated on the Illumina 450K platform (using the same pre-processing steps as in the “Power and prior sensitivity analysis” section).

The data used to estimate the computational time were generated as follows. First, we simulate each individual’s DNAm profile using independent and identically distributed (iid) uniform random variables on [0, 1]. More precisely, we simulate each individual’s DNAm as being generated by the Illumina 450K array, by simulating the value of each probe on the array using a random variable on [0, 1]. Next, we apply the same pre-processing steps as in the “Power and prior sensitivity analysis” section, resulting in 4731 regions to test for association.

Finally, in the “Computational cost” section of the Appendix, we derive the theoretical computational cost for WaveQTL and FFW.

### Type I error

**Fig. 2:**
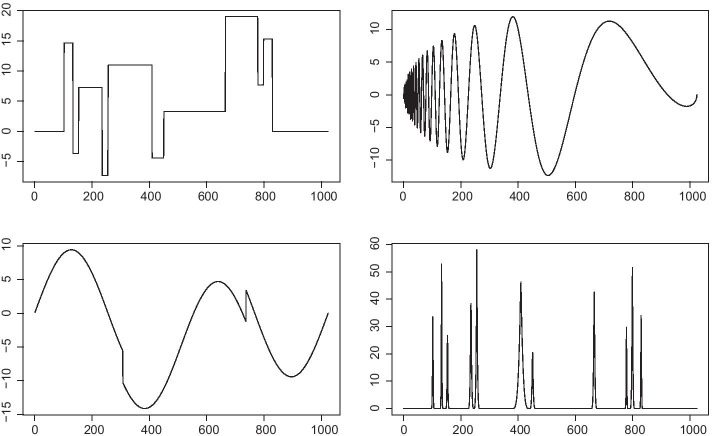
Test functions evaluated for type I error. Top left: Block; top right: Doppler; bottom left: HeaviSine; and bottom right: Bump

We estimated the type I error for four distinct scenarios and performed simulations under the assumption of no association, using the test functions *block*, *bump*, *heaviSine*, and *doppler* as previously described in Donoho and Johnstone [17]. The different functions are illustrated in Fig. 2 (adapted from Donoho and Johnstone [17]).

For each simulation *s*, we propose the following model of association. For each test function *T*, we perform the following simulation, and using *T*, we generate a population of observed functions as follows:

$$\begin{aligned} f_k(x) = a_k\times T(x)+ \epsilon \end{aligned}$$

where $a_k$ is the individual amplitude of the test function, with $a\sim N\left( 0,1 \right)$ and $\epsilon \sim N\left( 0,1 \right)$. We denote *F* as the set of observed functions. We then generate $Y_k \sim N\left( 0,1 \right)$, which represents a continuous trait not associated with the considered test function. Next, we wavelet-transform each individual function $f_k$ and quantile-transform each wavelet coefficient in the population. We then compute the likelihood ratio ${\hat{\Lambda }} ({\tilde{F}}, Y )_s$, as well as $\lambda _{1_s}$, which is the largest eigenvalue of $X\left( X^t X+ V_b ^{-1}\right) ^{-1}X^t$, where $X= \left( {\mathbf {1}},Y\right)$ and $V_b=diag( \sigma _b ^2 )$ ($\sigma _b$ is the prior effect size of the NIG prior for the intercept and the covariate). We simulate ${\hat{\Lambda }} ({\tilde{F}}, Y )_s$ $M=10^6$ times using

12 $$\begin{aligned} 2log(BF) \approx \lambda _{1_s} \chi ^2_1+ log(1-\lambda _{1_s}) \end{aligned}$$

These simulations are denoted as ${\hat{\Lambda }} ({\tilde{F}}, Y )^m_s$. We compute the Monte Carlo p value as

13 $$\begin{aligned} {\hat{p}}_s =\frac{ Card\left( m, {\hat{\Lambda }} ({\tilde{F}}, Y )_s \ge {\hat{\Lambda }} ({\tilde{F}}, Y )^m_s \right) +1}{M+1} \end{aligned}$$

This procedure is repeated 75,000 times for each sample size and type of test function (*block*, *bump*, *heaviSine*, and *doppler*). Table 1 summarizes the estimated type I errors for different sample sizes and test functions. These results show that the type I errors are handled satisfactorily for all the function types and sample sizes.

**Table 1:** Estimated type I error for different sample sizes and test functions

Test function	*n*	$\alpha$ level			
		0.0500	0.0100	0.0010	0.0001
Block	100	0.49980	0.00992	0.00111	0.00017
Block	500	0.50048	0.00978	0.00105	0.00009
Block	1000	0.49950	0.01028	0.00090	0.00014
Bump	100	0.50040	0.01026	0.00111	0.00004
Bump	500	0.50182	0.01040	0.00080	0.00009
Bump	1000	0.50050	0.00961	0.00098	0.00008
HeaviSine	100	0.50007	0.00982	0.00085	0.00009
HeaviSine	500	0.49956	0.00975	0.00091	0.00007
HeaviSine	1000	0.49965	0.01026	0.00103	0.00009
Doppler	100	0.50054	0.01014	0.00097	0.00009
Doppler	500	0.50089	0.01007	0.00100	0.00008
Doppler	1000	0.49916	0.01037	0.00095	0.00013

### Power and prior sensitivity analysis

To evaluate the power and performance of FFW on DNAm data, we used the same dataset as in Lee and Morris [5]. This dataset is a combination of 26 methylation profiles on chromosome 3, containing a total of 75,069 probes. Every patient’s methylation profile was measured twice, once in cancer cells and once in control cells. The phenotype (*Y*) is thus a binary indicator corresponding to a cancer (*Y* = 1) or control (*Y* = 0) cell.

The simulations are designed as follows. The true mean methylation level is kept identical for all the probes except the 1901 loci that were previously found to be differentially methylated in Irizarry et al. [18]. For these 1901 probes, the mean methylation levels were made to be different between cases and controls according to the difference reported by Irizarry and colleagues [18].

The above simulations are designed to ensure that the two groups have the same DNAm profile for all CpGs except the 1901 loci reported to be differentially methylated in Irizarry et al. [18]. For further information regarding the simulated data, readers are referred to the Supporting Information section in Lee and Morris [5].

The dataset itself is available at http://odin.mdacc.tmc.edu/~jmorris/simulated_data.Rdata.

*Pre-processing* As CpG sites are not evenly spaced in the genome, the wavelet transform is well-suited for modeling such sites as a function, provided there is a sufficiently large number of measurements. We pre-processed the DNAm data by dividing the genome into smaller regions containing at least 10 CpGs, with any two adjacent CpGs separated by a maximum distance of 500 base pairs. This criterion is similar to the one used by Jenkinson et al. [19], where the authors studied regions of 3 Kb containing at least 10 CpGs.

**Fig. 3:**
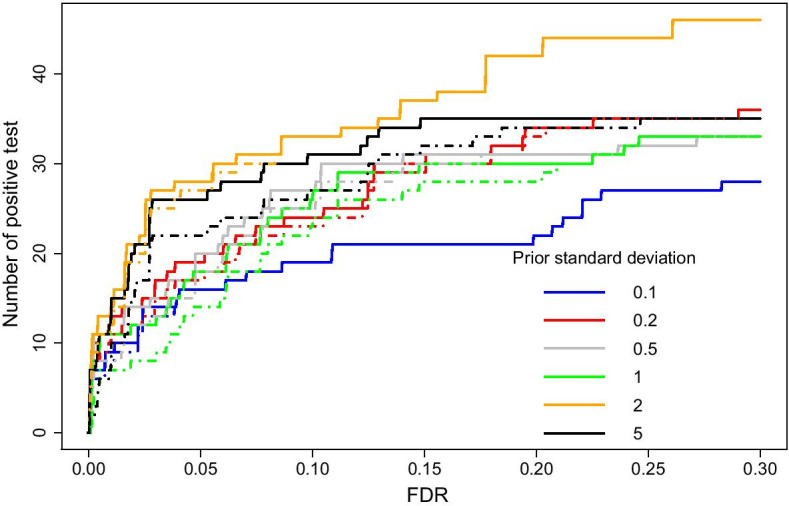
Assessing the sensitivity of the standard deviations of the prior. Each curve represents the number of DNAm regions detected at a given FDR and according to different standard deviations of the prior. The solid curves are the output of FFW; the dashed curves are the output of WaveQTL

The above pre-processing step resulted in a total of 1213 regions, containing 1875 of the 1901 CpGs in Irizarry et al. [18] that were scattered across 89 of the 1213 defined regions. For each region, we investigated whether the CpG patterns varied between case (*n* = 13) and control (*n* = 13) cells. As each region contains at least 10 CpGs, we used a depth of analysis of 3. We then ran FFW and WaveQTL for different values of the standard deviation of the prior on the previously defined regions. Finally, for each method and standard deviation of the prior, we computed the p value for each region and the corresponding false discovery rate (FDR) using the Benjamini–Hochberg procedure [20]. Figure 3 shows the consistency of FFW according to different standard deviations of the prior. This figure also shows that FFW and WaveQTL have similar power.

To evaluate the type I error of FFW for various standard deviations of the prior, we used the above dataset of cancer and control cells to generate the test statistics under the null. We performed 50 screenings of the dataset by permuting the phenotype in each screening. This corresponds to 56,500 observations of the test statistics under the null for different standard deviations of the prior. Table 2 summarizes the estimated type I errors for different standard deviations of the prior. The calibration is slightly worse than the one displayed in Table 1, which might be because the approximation used here is only valid asymptotically (see Eq. 11). Moreover, only 26 individuals were available for analysis in the case-control dataset, and we only considered independent samples in our modeling. Even though the dataset contained repeated measurements, the estimations were similar across the different priors. As was the case with statistical power, Fig. 3 also shows that FFW and WaveQTL have a similar performance.

**Table 2:** Estimated type I error for different standard deviations of the prior

Prior $\sigma _b$	$\alpha$ level			
	0.0500	0.0100	0.0010	0.0001
0.1	0.0416	0.0183	0.0016	0.0008
0.2	0.0451	0.0145	0.0019	0.0012
0.5	0.0451	0.0236	0.0020	0.0010
1	0.0478	0.0119	0.0019	0.0009
2	0.0500	0.0110	0.0017	0.0009
5	0.0522	0.0210	0.0017	0.0008

We assessed the power of FFW according to the number of differentially methylated CpGs per region (Table 3). We computed the average proportion of truly associated regions across different standard deviations of the prior for each FDR level and the number of differentially methylated CpGs per region. FFW had higher power for regions containing a large number of differentially methylated CpGs (Table 3). We also computed the power according to the number of differentially methylated CpGs per region using WaveQTL. We found the same power estimates as those shown in Table 3. This is unexpected, considering the relatively small number of truly associated regions (*n* = 89). Figure 3 also shows slight differences between FFW and WaveQTL in relation to FDR. Still, these discrepancies are negligible and indicate that the two approaches have similar power (Additional file 1). Figure 4 shows matching ROC curves for FFW and WaveQTL, which indicate that the two methods have the same power. We suspect that, since WaveQTL estimates the p value using an early-stopping Monte Carlo approximation based on 10,000 permutations, the estimated FDR might be slightly more conservative than the one obtained based on simulations. However, when we rank the regions by FDR, we obtained the same ranking for WaveQTL as for FFW. As ROC curves are invariant if the ranking of the regions does not change, we obtain the same ROC curves as a result.

**Table 3:** Estimated power according to the number of differentially methylated CpGs per region

FDR	Number of CpGs			
	1–10	11–20	21–30	$\ge$ 30
0.01	0.000	0.101	0.094	0.385
0.05	0.117	0.182	0.254	0.58
0.10	0.150	0.256	0.348	0.564
0.15	0.233	0.291	0.370	0.564
0.20	0.300	0.302	0.370	0.577

To compare FFW with another wavelet-based method, we repeated the same analyses using the “Wavelet-based Functional Mixed Models” (WFMM) method by Morris and Carrol [7] on the same dataset as above. WFMM can be used to detect DMRs [5], and, more generally, to detect signals via wavelet regression. The authors used an empirical Bayes approach to perform a regularization of the estimated effects. Their model can thus take into account a larger range of correlations between the observed DNAm profiles in each individual. WFMM is thus able to handle repeated measures of DNAm.

WFMM processed all the 75,069 CpG sites in one go and computed the posterior probability for each of the CpGs being above a set threshold (here 0.05) for being associated with cancer. We ran WFMM by specifying the correlation structure between the observations. Following the approach of Lee and Morris [5], we transformed the posterior probabilities of the CpGs into Bayesian FDR [5]. To compare the performance of WFMM against that of FFW, we first need to provide a regional significance criterion for WFMM. We used the minimum Bayesian FDR value for all the CpGs within a region of interest to assign a regional significance criterion. After running WFMM on the entire dataset, we used the minimum Bayesian FDR value for each of these regions to assess significance.

The results showed that WFMM had higher power than FFW, detecting all the 89 regions with an FDR below 0.01. This difference in power might be due to the refined modeling proposed by Morris and Carrol [7], which takes advantage of the correlations between DNAm profiles. Notably, Lee and Morris [5] showed that taking these correlations into account resulted in a systematic gain in power. In terms of computational time, however, WFMM took more than 6 h to complete the screening of 1213 regions, whereas FFW took a minute.

## Discussion

This paper reports on a computational shortcut for improving the wavelet-based approach proposed by Shim and Stephens [8]. We drew inspiration from the work of both Shim and Stephens [8] and Zhou and Guan [10] to develop a faster functional modeling that is applicable to a wider variety of functions and phenotypes. The approach of Shim and Stephens [8] was designed to identify dsQTL. Here, we show that wavelet-based approaches can also be used to detect differentially methylated regions (DMRs). Both WaveQTL by Shim and Stephens [8] and FFW offer a more flexible approach to modeling functions than conventional single-point testing. By keeping the design matrix constant across the screened regions and using simulations instead of permutations, we show that FFW is faster than WaveQTL. In addition, FFW controls the type I error satisfactorily for large sample sizes.

Reverse regression is a very useful tool for reducing the overall computational burden. However, the downside of reverse regression is that the coefficients from the analysis may become less interpretable. If the objective of a study is to estimate the effect of a particular wavelet in a given DNAm region, then one needs to rerun the procedure using individual wavelet coefficients as exposures. Therefore, FFW might function better as an initial screening tool to gain important biological insights from the DNAm data. For other types of data, such as those from biomechanical research, the wavelet coefficients are more directly interpretable. For example, if a researcher is interested in studying the effect of a particular treatment on motor function, e.g., leg function in the strength-dexterity test [21], FFW would lend itself easily to such an analysis.

An additional methodological constraint is the need to assign a given value for *J* in the applications of FFW. We chose a cutoff of *J* = 3 because of the requirement for the screened regions to contain least 10 CpGs, with any two adjacent CpGs separated by a maximum distance of 500 base pairs. In general, we advise choosing as large of a *J* as possible, while restricting $2^J < \kappa$, where $\kappa$ is an integer larger than 4. This can be written as $J=max _j\{ 2^j < \kappa \}$ when analyzing regions containing at least $\kappa$ CpGs. In our current application, this corresponded to *J* = 3. Nevertheless, it is possible to choose a different *J* for each region. Given the test statistic depends on *J*, choosing a different *J* for each genetic region would require the test statistic to be simulated for different values of *J*. As shown in Fig. 1, one can quickly simulate the test statistic. Therefore, simulating different values of *J* is likely to have a negligible impact on the overall run time.

FFW is well-powered to detect DMRs containing more than 10 CpGs, even when the CpGs only have small effects. However, FFW has lesser power for detecting DMRs containing only a few CpGs ($\le 10$). Although it is less powerful than WFMM [5], FFW has the advantage of being significantly faster in terms of computational time. WFMM took more than 6 h to process one chromosome for 26 individuals, whereas FFW took a minute. We thus expect WFMM to become exceedingly slow if there is a need to scale up the analysis to include hundreds of individuals and data from denser DNAm platforms, such as the Illumina 850K, or those from whole-genome bisulfite sequencing [22]. Therefore, FFW is a useful complementary tool for the rapid scanning of EWAS datasets to detect DMRs that can subsequently be used in downstream fine-mapping efforts. An attractive application of FFW would be to re-analyze DNAm data from previously published EWASes that are publicly available through, e.g., the Gene Expression Omnibus (GEO) database [23].

In future developments, we plan to extend FFW to also include phenotypes on non-ordered scales, e.g., blood types and psychiatric phenotypes. Such phenotypes are routinely treated in a case-control fashion and analyzed using multinomial regression owing to the prohibitively large computational burden. However, by exploiting reverse regression, as we do here, the phenotypes can be re-coded and readily included in the predictor matrix. Reverse regression also enables FFW to easily adapt to the setting of a phenome-wide association study (PheWAS), in which multiple phenotypes are interrogated simultaneously ([24–26]). As highlighted by our analyses, this development is further simplified by the results of Zhou and Guan [10], showing that the parameter of the Bayes factors law depends primarily on the singular values of the regression matrix and the number of parameters tested. As the regression matrix remains constant across all loci, locations, and scales, these parameters only need to be computed once, thus enabling a fast computation of p values. This makes FFW a highly versatile method for analyzing phenotypes that do not lend themselves easily to either single-point or bump-hunting methods.

FFW is distributed as an ***R*** package. The package contains the analysis code and a data visualization tool to enable a more detailed inspection of the detected regions. The full ***R*** package is freely available on GitHub (https://github.com/william-denault/ffw), and a comprehensive example run of the package is provided in the help function *ffw*.

## Supplementary Information

**Additional file 1: Figure S4**. ROC curves at given standard deviations of the prior. The thin solid curves are the output of FFW; the thick dashed curves are the output of WaveQTL. The ROC curves match for standard deviations of the prior of 0.5 and 1.

## Data Availability

The full R package for FFW is freely available on GitHub (https://github.com/william-denault/ffw), and a comprehensive example run of the package is provided in the help function *ffw*. The data supporting the findings of this study are openly available at http://odin.mdacc.tmc.edu/~jmorris/simulated_data.Rdata.

## Appendix

### Computational cost

Here, we discuss the improvement in computational cost using FFW. Let *R* be the number of regions to screen for associations (i.e., the number of tests to be performed). When considering a Bonferroni correction for *R* tests, the number of permutations or simulations needed to obtain sufficiently precise p values for a multiple testing correction of $\frac{0.05}{R}$ is of the order $O\left( R^2\right)$ (see Knijnenburg et al. [27]). The computational cost of performing a linear regression is $O\left( np^2+p^3\right)$, where *n* is the number of observations and *p* is the number of variables. For every screened region, we perform $2^J$ linear regressions. The complexity of the wavelet transform is equal to the number of observed variables, which is $2^J$ in our case. It follows that the combined computational cost using WaveQTL for a full screening of *R* regions and the permutation procedure is:

14 $$\begin{aligned} O\left( 2^{J} \left( np^2+p^3 +e \right) \left( 1+R^2\right) R+ 2^{J}R\right) \end{aligned}$$

where *e* is the average number of iterations of the EM algorithm. Using early-stopping methods described by Gandy [28] and Besag and Clifford [29] for deriving Monte Carlo p values, the computational cost can be further reduced to:

15 $$\begin{aligned} O\left( 2^{J} \left( np^2+p^3 +e \right) \left( 1+S\right) R+ 2^{J}R\right) \end{aligned}$$

where *S* is the expected number of steps of the early-stopping Monte Carlo p values method, with $S \le R^2$. The first term in Eq. (15) is the number of regressions to be performed, including the ones for the permutation as well as for the cost of the EM algorithm. The second term is the overall cost of the wavelet transform.

Early-stopping methods, such as the methods of Gandy [28] or Besag and Clifford [29], are algorithms that estimate p values in a sequential fashion. As in most applications, the interesting p values are the small p values. Early-stopping methods aim at avoiding using a lot of computational power on large p values. Therefore, when it is likely that the test p value is large, such algorithms stop and move on to the next p value to be estimated. Early-stopping methods thus aim at focusing only on the interesting tests and allocating most of the computational power to those. The *simctest* *R* package contains most of recent early-stopping algorithms, and include the method of Gandy [28].

Using FFW, and neglecting the cost of simulations, we obtain a computational cost of:

16 $$\begin{aligned} O\left( 2^{J} \left( np^2+p^3 \right) R+ 2^{J}R +e\left( R+R^2\right) \right) \end{aligned}$$

where the first term is the computational cost of all the regressions performed, the second term is the overall cost of the wavelet transform, and the third term is the number of iterations performed by the EM algorithm. The *R* term is for running the EM algorithm for each region, and the $R^2$ term is for running the EM algorithm for each simulation used to assess the significance of each region. Using early-stopping rules for Monte Carlo p values, this can be further reduced to:

17 $$\begin{aligned} O\left( 2^{J} \left( np^2+p^3 \right) R+ 2^{J}R +e\left( R+S'\right) \right) \end{aligned}$$

where $S'$ is the number of iterations needed to assess the significance of the smallest p value using the early-stopping rules for Monte Carlo p values ($S' \le R^2$). In essence, this procedure reduces the degree of the polynomial cost of the region by one. In addition, the term $\left( np^2+p^3\right)$, which carries part of the computational burden, is now only associated with a first-order polynomial in *R* and no longer associated with a third and second-degree polynomial. In a scenario in which all the regions have $2^J$ variables, the first term would thus represent the overall cost corresponding to the single-point testing strategy.

## Abbreviations

DMR: Differentially methylated region
DNAm: DNA methylation
dsQTL: DNase I hypersensitivity quantitative trait locus
EWAS: Epigenome-wide association study
FDR: False discovery rate
GEO: Gene Expression Omnibus
GWAS: Genome-wide association study
MCMC: Markov Chain Monte Carlo
NIG: Normal-inverse gamma
PheWAS: Phenome-wide association study
QTL: Quantitative trait locus
