# Correction to: Detecting differentially methylated regions using a fast wavelet-based approach to functional association analysis

**DOI:** 10.1186/s12859-021-04160-1

**Published:** 2021-05-26

**Authors:** William R. P. Denault, Astanand Jugessur

**Affiliations:** 1 Department of Genetics and Bioinformatics, Norwegian Institute of Public Health, Oslo, Norway; 2 Centre for Fertility and Health, Norwegian Institute of Public Health, Oslo, Norway; 3 Department of Global Public Health and Primary Care, University of Bergen, Bergen, Norway

## Correction to:BMC Bioinformatics (2021) 22:61 10.1186/s12859-021-03979-y

Following publication of the original article [1], the authors identified an error in Table 1. The correct Table 1 is given below.

**Table 1:** Estimated type I error for different sample sizes and test functions

Test function	*n*	$\alpha$ level			
		0.5000	0.0100	0.0010	0.0001
Block	100	0.49980	0.00992	0.00111	0.00017
Block	500	0.50048	0.00978	0.00105	0.00009
Block	1000	0.49950	0.01028	0.00090	0.00014
Bump	100	0.50040	0.01026	0.00111	0.00004
Bump	500	0.50182	0.01040	0.00080	0.00009
Bump	1000	0.50050	0.00961	0.00098	0.00008
HeaviSine	100	0.50007	0.00982	0.00085	0.00009
HeaviSine	500	0.49956	0.00975	0.00091	0.00007
HeaviSine	1000	0.49965	0.01026	0.00103	0.00009
Doppler	100	0.50054	0.01014	0.00097	0.00009
Doppler	500	0.50089	0.01007	0.00100	0.00008
Doppler	1000	0.49916	0.01037	0.00095	0.00013

The author group has been updated above and the original article [1] has been corrected.
