# Supplementary figures and images for: Detecting differentially methylated regions using a fast wavelet-based approach to functional association analysis
Source: BMC Bioinformatics. 2021 Feb 10;22:61. doi: 10.1186/s12859-021-03979-y (PMC7876806; doi:10.1186/s12859-021-03979-y)

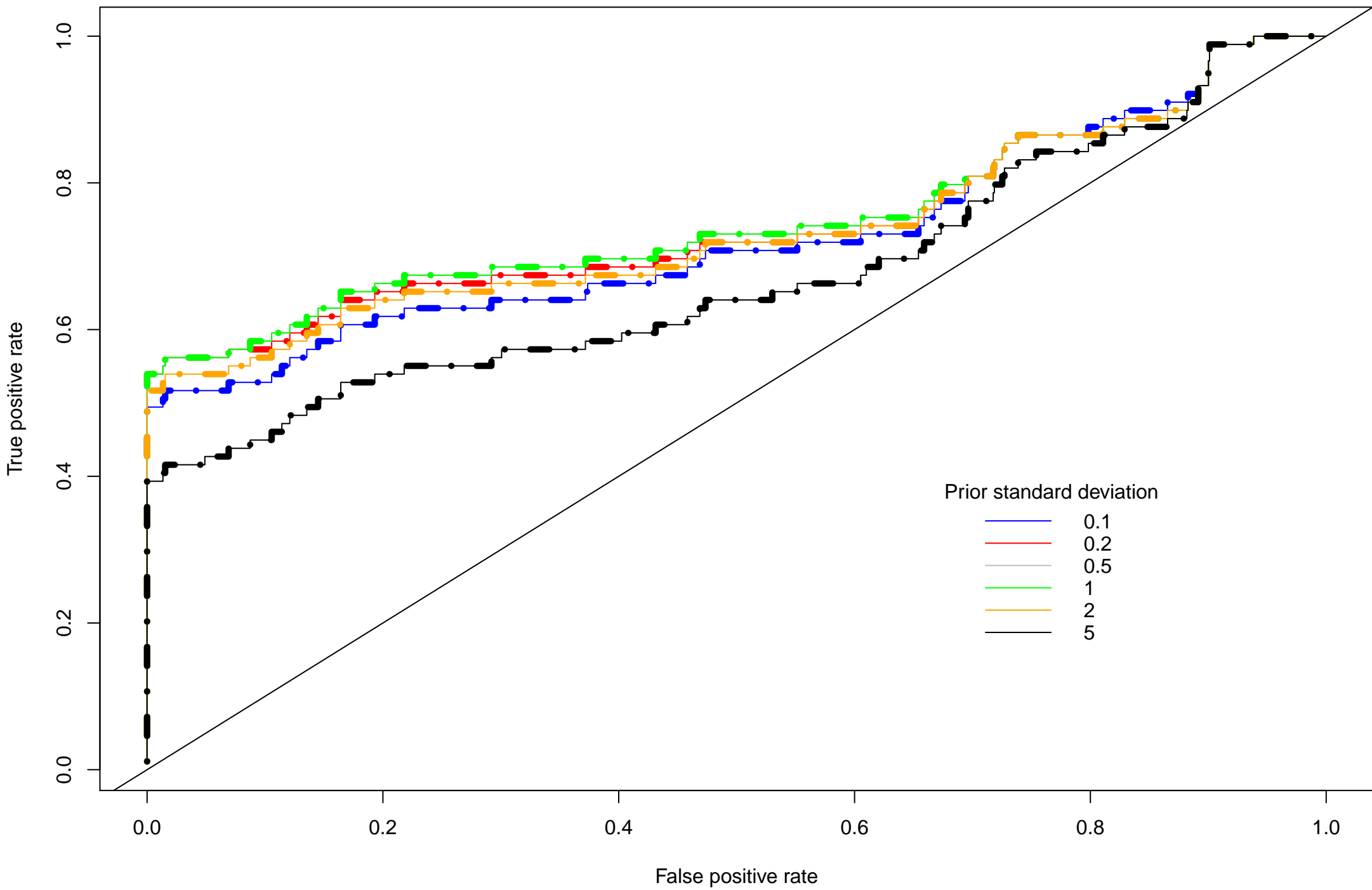

Supplement: Supplementary file 1 — Additional file 1: Figure S4. ROC curves at given standard deviations of the prior. The thin solid curves are the output of FFW; the thick dashed curves are the output of WaveQTL. The ROC curves match for standard deviations of the prior of 0.5 and 1. [file 12859_2021_3979_MOESM1_ESM.pdf]
